# The relationship between post-traumatic stress disorder, occupational stress, occupational burnout, and mental health in football referees: a national cross-sectional survey in China

**DOI:** 10.3389/fpubh.2025.1647115

**Published:** 2025-09-08

**Authors:** Liangyu Zhao, Liguo Zhang

**Affiliations:** School of Physical Education, Shandong University, Jinan, China

**Keywords:** post-traumatic stress disorder, mental health, occupational stress, occupational burnout, football referees

## Abstract

**Background:**

The SARS-CoV-2 pandemic, as a global public health emergency, is widely recognized as a major traumatic event with far-reaching implications for individual mental health. Football referees, as a distinct occupational group, may have been particularly vulnerable to elevated trauma exposure and psychological burden during the pandemic. However, research on the post-pandemic mental health status of this population—and the mechanisms underlying it—remains scarce. This study aims to investigate the relationships and underlying mechanisms among post-traumatic stress disorder (PTSD), occupational stress, occupational burnout, and mental health among Chinese football referees in the aftermath of the SARS-CoV-2 outbreak.

**Methods:**

This cross-sectional study surveyed 344 football referees across 29 provinces in China using standardized questionnaires, including the Impact of Event Scale-Revised (IES-R), Depression Anxiety Stress Scale (DASS-21), Effort–Reward Imbalance Questionnaire (ERI), and the Maslach Burnout Inventory–General Survey (MBI-GS). Data were analyzed using network analysis, exploratory structural equation modeling (ESEM), and bootstrapped mediation testing.

**Results:**

The prevalence of moderate-to-severe PTSD among football referees was 26.2%, with PTSD significantly negatively predicting mental health outcomes. Network analysis identified effort and emotional exhaustion as central and bridging dimensions across variables. The set-ESEM supported the network analysis findings and demonstrated good model fit. Mediation analyses revealed that occupational stress and occupational burnout served as both independent and sequential mediators in the relationship between PTSD and mental health. Specifically, effort, overcommitment, emotional exhaustion, and cynicism played pivotal roles in this mechanism, while reward and professional efficacy did not exhibit significant mediating effects.

**Conclusion:**

This study confirms the applicability of the job demands–resources model and stressor theories, highlighting occupational stress and occupational burnout as critical mediating mechanisms between PTSD and mental health in football referees. These findings provide theoretical and empirical foundations for public health interventions aimed at improving the psychological well-being of referees.

## 1 Introduction

The emergence of post-traumatic stress disorder (PTSD) and adverse mental health outcomes following infectious disease pandemics has garnered widespread attention. Previous public health emergencies—such as Severe Acute Respiratory Syndrome (SARS), Ebola virus disease, and Middle East Respiratory Syndrome Coronavirus (MERS-CoV)—have all been empirically linked to elevated risks of PTSD (1–3). As a global public health crisis, the SARS-CoV-2 pandemic has likewise been recognized as a traumatic event, resulting in over 7.0 million deaths and infecting more than 770 million individuals worldwide (4). Evidence indicates that ongoing exposure to infection risks, illness, and mortality during the pandemic has triggered acute stress responses, giving rise to PTSD symptoms. A systematic review estimated the post-pandemic prevalence of PTSD at approximately 22.6% (5), while other studies report PTSD symptoms in nearly 42% of SARS-CoV-2 survivors (6). Moreover, the social isolation and work suspensions induced by the pandemic generated significant socio-economic uncertainty. The virus’s high transmissibility and mortality rate further provoked widespread fear, anxiety, and depression, collectively exerting detrimental effects on public mental health (7). The impact was particularly severe for outdoor workers, such as football referees, who faced disruptions in their professional activities, unstable incomes, and declines in physical fitness due to limited training opportunities.

As of 2014, China had registered approximately 2,000 football referees (8), with only 32 additional international-level referees added by 2023 (9). Although the number of referees remains relatively small, their role in competitive sports is indispensable. By enforcing rules and overseeing gameplay, referees ensure fairness and integrity in matches (10). This occupation has distinctive features: referees must withstand split-second decision pressure during matches, submit to media oversight and scrutiny, and bear public accountability. These characteristics place refereeing within a context of heightened social-evaluative threat and responsibility, which may amplify stress responses and emotional demands beyond those typically observed in other occupations. During the SARS-CoV-2 pandemic, the postponement and suspension of sporting events in China significantly reduced referees’ income. Simultaneously, a lack of physical activity led to declines in physical fitness and professional competence (11), potentially exacerbating adverse mental health outcomes. Therefore, investigating the prevalence and interrelations of PTSD, occupational stress, occupational burnout, and mental health among football referees in the aftermath of the SARS-CoV-2 pandemic is not only of theoretical significance but also has practical implications for the expansion of the referee workforce and the broader development of football in China. Hence hypothesis is proposed:

*Hypothesis 1*: PTSD negatively impacts the mental health of football referees.

The PTSD is a psychiatric condition triggered by acute traumatic events or natural disasters, characterized by symptoms such as intrusive recollections, heightened arousal, avoidance behaviors, and emotional numbing (12). Emerging evidence suggests that the SARS-CoV-2 pandemic significantly reduced individuals’ levels of physical activity and physical fitness, leading to post-recovery concerns regarding personal health status and occupational competence, thereby intensifying occupational stress (13). Occupational stress refers to the physiological and psychological strain experienced when job demands exceed an individual’s adaptive capacity (14). Based on the Effort-Reward Imbalance (ERI) model, occupational stress comprises three dimensions: effort (the demands and obligations of the job), reward (monetary, self-esteem, and career-related rewards), and overcommitment (a coping pattern reflecting excessive effort and difficulty disengaging from work). Research has found that PTSD impairs attentional control and increases appraisal of external threat, thereby elevating perceived effort and imbalance—especially under split-second decision pressure, where cognitive load and time scarcity constrain self-regulation. Prolonged occupational stress increases the risk of mental illness. In addition, lockdowns and widespread business closures during the pandemic precipitated an economic downturn. Subsequently, many organizations implemented layoffs and salary reductions to relieve financial pressure, further exacerbating workers’ psychological stress and anxiety. According to the stressor theory, perceived stress can lead to various mental health problems, including anxiety and depression (15). For football referees, physical fitness and technical proficiency are essential for professional performance and career progression. The suspension of all sporting events during the SARS-CoV-2 pandemic significantly curtailed their physical activity and diminished their physical conditioning. At the initial stage of competition resumption, lapses in professional proficiency likely increased referees’ occupational stress, especially given concerns over their ability to quickly readapt to the demands of high-intensity officiating (16). Prolonged occupational stress is strongly associated with the onset of anxiety, depression, and other adverse psychological symptoms, ultimately compromising referees’ overall mental health (17). Based on this, the following hypotheses are proposed:

*Hypothesis 2*: Occupational stress mediates the relationship between PTSD and mental health.

Occupational burnout is a psychological syndrome stemming from chronic work-related stress and is characterized by three core dimensions: emotional exhaustion (feelings of being emotionally drained), cynicism (detachment and negative attitudes toward work), and reduced professional efficacy (feelings of incompetence and lack of achievement) (18). According to the Job Demands–Resources model, when job demands significantly or persistently exceed available resources, it can lead to a range of negative outcomes, such as reduced work performance and diminished organizational commitment. The sustained depletion of personal energy may ultimately result in burnout, with adverse consequences for both physical and mental health (19). Specifically, upon returning to the field following the SARS-CoV-2 pandemic, football referees faced increased job demands due to intensified work schedules, fewer employment opportunities, and reduced salaries. These heightened demands were not offset by additional job resources, thereby increasing the risk of occupational burnout. Notably, occupational burnout has long been a critical concern among football referees. On one hand, they are frequently subjected to verbal abuse from players, coaches, and spectators, intense scrutiny from the media, and the inherent demands of the role itself (20). On the other hand, their work seldom receives recognition or appreciation, and they are often subject to public scrutiny and accountability. Such conditions may lead referees to lose interest and motivation in their profession, thereby fostering occupational burnout (21). Prolonged occupational burnout continuously depletes referees’ energy reserves, diminishes cognitive responsiveness, and reduces work efficiency. These effects may contribute to the development of anxiety and depressive symptoms, ultimately undermining mental health (22). Therefore, occupational burnout may mediate the relationship between PTSD and mental health outcomes. Based on this, the following hypotheses are proposed:

*Hypothesis 3*: Occupational burnout mediates the relationship between PTSD and mental health.

Substantial evidence across various sectors indicates that occupational stress is a significant predictor of occupational burnout (23). According to the Job Demands–Resources model, when individuals are subjected to sustained high levels of work-related stress—such as excessive workload, task complexity, and role conflict—without access to adequate resources like self-efficacy or social support, occupational burnout is likely to ensue (19). During the SARS-CoV-2 pandemic and subsequent lockdowns, football referees experienced a decline in technical proficiency, physical fitness, endurance, and responsiveness. Upon returning to their roles in a high-intensity and high-pressure environment, they faced elevated job demands, which likely increased perceived occupational stress. On one hand, football seasons typically span extended periods; on the other hand, referees require time to regain pre-pandemic levels of fitness and technical skill. Such prolonged exposure to occupational stress may gradually evolve into occupational burnout, manifesting in reduced work efficiency, heightened anxiety and depressive symptoms, and compromised mental health (24). Based on the Job Demands–Resources model and existing empirical evidence, this study treats occupational stress as a precursor to occupational burnout, and both as mediators in the pathway from PTSD to mental health. Therefore, hypothesis is proposed:

*Hypothesis 4*: Occupational stress and occupational burnout serve as sequential mediators in the relationship between PTSD and mental health.

In summary, existing research on the relationships among PTSD, occupational stress, occupational burnout, and mental health has largely focused on healthcare workers and athletes, with minimal attention paid to football referees. However, given the distinctive nature of refereeing, there is an urgent need to empirically verify the applicability of the Job Demands–Resources model and stressor theory in this group and thereby broaden their theoretical scope. Therefore, grounded in the Job Demands–Resources model and stressor theory, this study explores the multidimensional interplay between PTSD, occupational stress, occupational burnout, and mental health. The findings aim to provide theoretical guidance for improving referees’ psychological well-being and informing public health interventions.

## 2 Method

### 2.1 Participants and procedure

This study employed a cross-sectional design using simple random sampling. Participants were certified football referees officially recognized by the national governing body, representing 29 provinces across China. Data collection took place between September and October 2022 during the 14th National Student Sports Games of the People’s Republic of China, held in Qingdao, Shandong Province. Participants were recruited on-site during the event and invited to participate voluntarily. They were given the option to complete the questionnaire either (1) through a face-to-face, interview-administered survey conducted by trained research assistants, or (2) via an online platform (Wenjuanxing, https://www.wjx.cn/) accessed through an invitation link. This dual-mode administration was adopted to maximize participation, particularly among referees with scheduling or mobility constraints. Both modes employed an identical, structured questionnaire and were entirely quantitative in nature. The questionnaire captured demographic characteristics, the perceived impact of the COVID-19 pandemic, occupational stress, occupational burnout, and mental health status. All participants were informed of the anonymity and confidentiality of the study and provided written informed consent. A total of 356 questionnaires were collected. After excluding responses with completion times under 20 min or with missing data, 344 valid questionnaires remained, yielding a response rate of 96.6%. The study was approved by the Ethics Committee of Shandong University (ECBMSSDU2022-1-086).

### 2.2 Measures

#### 2.2.1 Post-traumatic stress disorder

The Chinese version of the Impact of Event Scale – Revised (IES-R) was used to assess referees’ psychological responses and traumatic experiences following the COVID-19 pandemic and associated lockdowns (25). The IES-R comprises 22 items, divided into three subscales: Avoidance, Intrusion, and Hyperarousal. Responses were rated on a 5-point Likert scale: 0 = “Not at all,” 1 = “Rarely,” 2 = “Sometimes,” 3 = “Often,” and 4 = “Always.” Total scores range from 0 to 88, with severity interpreted as follows: 0–8 (subclinical), 9–25 (mild), 26–43 (moderate), and 44–88 (severe). In this study, the IES-R demonstrated excellent internal consistency (Cronbach’s *α* = 0.952, CR = 0.955 > 0.7).

#### 2.2.2 Mental health

The Depression Anxiety Stress Scales – 21 (DASS-21) was used to evaluate participants’ emotional states of depression, anxiety, and stress over the past week (26). It includes three subscales—each with 7 items—for a total of 21 items. Responses are rated on a 4-point Likert scale: 0 = “Does not apply,” 1 = “Sometimes applies,” 2 = “Often applies,” and 3 = “Always applies.” Subscale scores are doubled to yield a final score, with each subscale ranging from 0 to 42 and the total scale from 0 to 126; higher scores indicate greater symptom severity. Scores within the non-clinical reference range are defined as: Depression ≤9, Anxiety ≤7, and Stress ≤14. In this study, the internal consistency coefficients for the Depression, Anxiety, Stress subscales and the total scale were 0.904, 0.890, 0.910, and 0.962, respectively.

#### 2.2.3 Occupational stress

The Effort-Reward Imbalance Questionnaire (ERI), developed by German sociologist Johannes Siegrist, was employed to assess the imbalance between effort and reward in the workplace (27). According to the ERI model, an imbalance between high effort and low reward may lead to elevated occupational stress. The scale includes three components: Effort (6 items), Reward (11 items), and Overcommitment (6 items), for a total of 23 items. The Effort and Reward subscales are rated on a 5-point Likert scale, with responses as follows: 1 = “Strongly disagree,” 2 = “Disagree, not troubling,” 3 = “Agree, somewhat troubling,” 4 = “Agree, troubling,” and 5 = “Agree, very troubling.” The Overcommitment subscale uses a 4-point Likert scale: 1 = “Strongly disagree,” 2 = “Disagree,” 3 = “Agree,” and 4 = “Strongly agree.” The Effort–Reward Imbalance Ratio (ERIratio) is calculated as Effort (E) ÷ Reward (R) × 0.54, with higher values indicating greater occupational stress. In the current study, the ERI demonstrated acceptable internal consistency (Cronbach’s *α* = 0.688, CR = 0.915 > 0.7).

#### 2.2.4 Occupational burnout

The Maslach Burnout Inventory – General Survey (MBI-GS), developed by Maslach and Jackson, is widely used to assess occupational burnout (18). Its Chinese version has demonstrated sound reliability and validity (28). This study utilized the 22-item MBI-GS, which includes three subscales: Emotional Exhaustion (8 items), Professional Efficacy (8 items), and Cynicism (6 items). The MBI-GS is broadly applicable across different occupational groups. Items are rated on a 5-point Likert scale: 1 = “Never,” 2 = “Rarely,” 3 = “Uncertain,” 4 = “Sometimes,” and 5 = “Always.” Items 9 through 16 are reverse-scored, while the remaining items are scored normally. The overall occupational burnout score is calculated by averaging the total item scores, with higher scores indicating greater occupational burnout severity. In this study, the MBI-GS showed good internal consistency (Cronbach’s *α* = 0.855, CR = 0.846 > 0.7).

### 2.3 Statistical analysis

#### 2.3.1 Descriptive statistics

All statistical analyses were conducted using IBM SPSS Statistics 26.0, Mplus, and R 4.3.2. Descriptive statistics were first performed for all variables. Continuous variables are presented as means ± standard deviations, while categorical variables are reported as frequencies and percentages. Group differences and correlations between variables were assessed using analysis of variance (ANOVA), chi-square tests, and Pearson correlation analysis. Harman’s single-factor test was used to assess potential common method bias. All statistical tests were two-tailed, and a *p*-value < 0.05 was considered statistically significant.

#### 2.3.2 Network analysis

Network analysis was conducted using the R packages qgraph, bootnet, and networktools to identify key nodes and bridge symptoms among PTSD, occupational stress, occupational burnout, and mental health indicators (29). The specific analytical procedures were as follows: (1) Network Estimation: The network was estimated using the graphical least absolute shrinkage and selection operator (GLASSO) method via the EBICglasso function in the bootnet package. The regularization parameter (*λ*) was set at 0.25 to balance model sparsity and estimation accuracy. (2) Centrality and Expected Influence: Centrality indices—strength, betweenness, closeness, and expected influence—were calculated using qgraph. Expected influence was prioritized for interpretation due to its suitability in networks containing both positive and negative edges, offering more nuanced insights into symptom interconnectivity. (3) Bridge Symptom Identification: The networktools package was used to compute bridge strength and bridge expected influence via its bridge function. Symptoms that linked distinct symptom clusters were identified and visualized to understand cross-domain interactions [0]. Nodes with higher bridge expected influence values indicated a greater likelihood of symptom transmission across conceptual domains. (4) Network Stability Assessment: The robustness of the network structure was evaluated using case-drop bootstrapping. This procedure assessed the stability and reliability of centrality indices and estimated confidence intervals (CIs) for edge weights. A narrower CI indicates greater precision. The centrality stability coefficient was calculated; a value above 0.25 indicates acceptable stability, and above 0.50 denotes strong robustness. (5) Network Comparison Test: The NetworkComparisonTest package (with 1,000 permutations) was used to assess differences between the original network and networks adjusted for covariates, as well as to test for gender-based differences. This included comparisons of global network strength, edge weights, and centrality indices. (6) Covariate-Adjusted Network Analysis: To control for potential confounding effects of demographic variables (age, gender, referee certification level, years of experience, smoking, and alcohol use), symptom scores were residualized via regression. The residualized symptom data were then analyzed using the EBICglasso method to isolate the intrinsic relationships among symptoms independent of covariate effects.

Network analysis offers an exploratory visualization of the structural interrelationships among PTSD, occupational stress, occupational burnout, and mental health dimensions. It facilitates the identification of key symptoms that bridge distinct domains and reveals potential targets for psychological intervention. However, as network models are inherently non-directional, they do not permit causal inference. Therefore, we subsequently employed exploratory structural equation modeling (ESEM) to validate the latent structure of the measured constructs and assess the model’s structural validity. Structural equation modeling (SEM) was then used to examine the directional relationships among PTSD, occupational stress, occupational burnout, and mental health. Finally, mediation analyses were conducted to evaluate both the independent and sequential mediating roles of occupational stress and occupational burnout in the relationship between PTSD and mental health.

#### 2.3.3 Set exploratory structural equation modeling (set-ESEM)

Given the complexity of the data structure—characterized by multiple latent constructs and potential cross-loadings—traditional confirmatory factor analysis (CFA) may be insufficient for accurately capturing the underlying structure. Therefore, this study employed set-ESEM, which integrates the strengths of ESEM and conventional SEM. This approach allows indicators to load freely on multiple latent variables, thereby providing a more flexible and realistic representation of the data’s latent structure (30). Model estimation was conducted using Mplus software. Unlike CFA, the set-ESEM specification did not constrain indicators to load on a single factor; instead, it permitted cross-loadings across multiple constructs. Parameters were estimated using the maximum likelihood method with robust standard errors to enhance the reliability of factor loadings and fit indices. Model fit was evaluated using conventional indices, including the Root Mean Square Error of Approximation (RMSEA), the Standardized Root Mean Square Residual (SRMR), *p*-values, and the 90% CI. Acceptable model fit was defined as RMSEA and SRMR values ≤ 0.08, a *p*-value < 0.05, and a 90% CI that does not include zero.

#### 2.3.4 Structural equation modeling (SEM)

To examine the directional pathways among PTSD, occupational stress, occupational burnout, and mental health, SEM was employed. Within the framework of the Job Demands–Resources model, PTSD was specified as the exogenous variable, occupational stress and occupational burnout as sequential mediators, and mental health as the outcome variable. Model estimation and assessment of fit indices were conducted using Mplus.

#### 2.3.5 Mediation analysis

Mediation effects of occupational stress and occupational burnout—as well as their respective subdimensions—were tested using PROCESS models 4 and 6 in SPSS. All mediation analyses were conducted with 5,000 bootstrap samples to generate bias-corrected 95% confidence intervals. A mediation effect was considered statistically significant if the confidence interval did not include zero.

## 3 Results

### 3.1 Descriptive analysis and correlation testing

Among the 344 participants, 290 were male referees (84.3%), and 113 held a Level 1 referee certification (32.9%). The majority had less than 3 years of officiating experience (36.6%). A total of 72 participants (20.9%) reported smoking, and 152 (44.2%) reported alcohol consumption. Table 1 presents the demographic and psychological characteristics of participants stratified by PTSD symptom severity. Compared with those exhibiting subclinical PTSD symptoms, referees with mild, and moderate and severe PTSD reported significantly higher scores in occupational stress, occupational burnout, stress, anxiety, and depression (*p* < 0.01). Correlation analysis revealed statistically significant associations among all variables except for reward and professional efficacy, which did not show significant correlations with other measures (*p* < 0.05; see Supplementary Table 1). Harman’s single-factor test indicated that 14 factors had eigenvalues greater than 1, and the first factor accounted for 29.86% of the total variance—below the 40% threshold—suggesting that common method bias was not a serious concern.

**Table 1:** Participant characteristics stratified by PTSD severity (*N* = 344).

Characteristics	PTSD			*Χ*^2^/*F*	*p*
	Subclinical	Mild	Moderate and severe		
Sex (*n*, %)					
Male	80 (27.6)	133 (45.9)	77 (26.6)	0.332	0.718
Female	13 (24.1)	28 (51.9)	13 (24.1)		
Age (*n*, %)					
≤18 years	2 (16.7)	5 (41.7)	5 (41.7)	0.867	0.544
19–25 years	45 (28.3)	79 (49.7)	35 (22.0)		
26–35 years	25 (27.8)	41 (45.6)	24 (26.7)		
36–45 years	19 (27.9)	30 (44.1)	19 (27.9)		
46 years and over	2 (13.3)	6 (40.0)	7 (46.7)		
Referee grade (*n*, %)					
International referee	0 (0.0)	1 (0.4)	0 (0.0)	2.300	0.018*
National referee	8 (17.8)	23 (51.1)	14 (31.1)		
Level 1 referee	20 (17.7)	53 (46.9)	40 (35.4)		
Level 2 referee	37 (34.6)	48 (44.9)	22 (20.6)		
Level 3 referee	28 (35.9)	36 (46.2)	14 (17.9)		
Working year (*n*, %)					
<3 years	39 (31.0)	60 (47.6)	27 (21.4)	1.678	0.122
3–5 years	25 (30.9)	40 (49.4)	16 (19.8)		
6–8 years	12 (26.1)	18 (39.1)	16 (34.8)		
≥8 years	17 (18.7)	43 (47.3)	31 (34.1)		
Smoking (*n*, %)					
No	72 (26.5)	130 (47.8)	70 (25.7)	0.259	0.772
Yes	21 (29.2)	31 (43.1)	20 (27.8)		
Drinking (*n*, %)					
No	51 (26.6)	90 (46.9)	51 (26.6)	0.032	0.969
Yes	42 (27.6)	71 (46.7)	39 (25.7)		
Occupational stress	0.18 ± 0.07	0.21 ± 0.09	0.27 ± 0.15	19.756	0.000**
Effort	10.10 ± 3.40	11.45 ± 3.68	14.30 ± 4.73	27.783	0.000**
Reward	31.13 ± 3.03	30.98 ± 3.39	30.43 ± 4.62	0.945	0.390
Overcommitment	12.27 ± 3.20	13.47 ± 2.82	14.71 ± 2.95	15.563	0.000**
Occupational burnout	1.67 ± 0.48	1.93 ± 0.51	2.21 ± 0.60	24.159	0.000**
Emotional exhaustion	13.83 ± 4.47	16.83 ± 5.61	20.59 ± 6.28	34.478	0.000**
Professional efficacy	13.34 ± 9.27	14.15 ± 8.99	13.79 ± 7.14	0.259	0.772
Cynicism	9.52 ± 4.03	11.45 ± 4.29	14.23 ± 4.97	26.491	0.000**
Mental health	3.59 ± 8.01	11.32 ± 14.30	27.60 ± 22.27	57.244	0.000**
Stress	1.42 ± 3.52	4.86 ± 5.70	10.44 ± 8.26	52.334	0.000**
Anxiety	1.12 ± 2.24	3.38 ± 4.67	8.58 ± 7.75	49.796	0.000**
Depression	1.05 ± 2.79	3.08 ± 4.88	8.58 ± 7.63	49.673	0.000**

*P < 0.05, **P <0.01.

### 3.2 Network analysis

**Figure 1:**
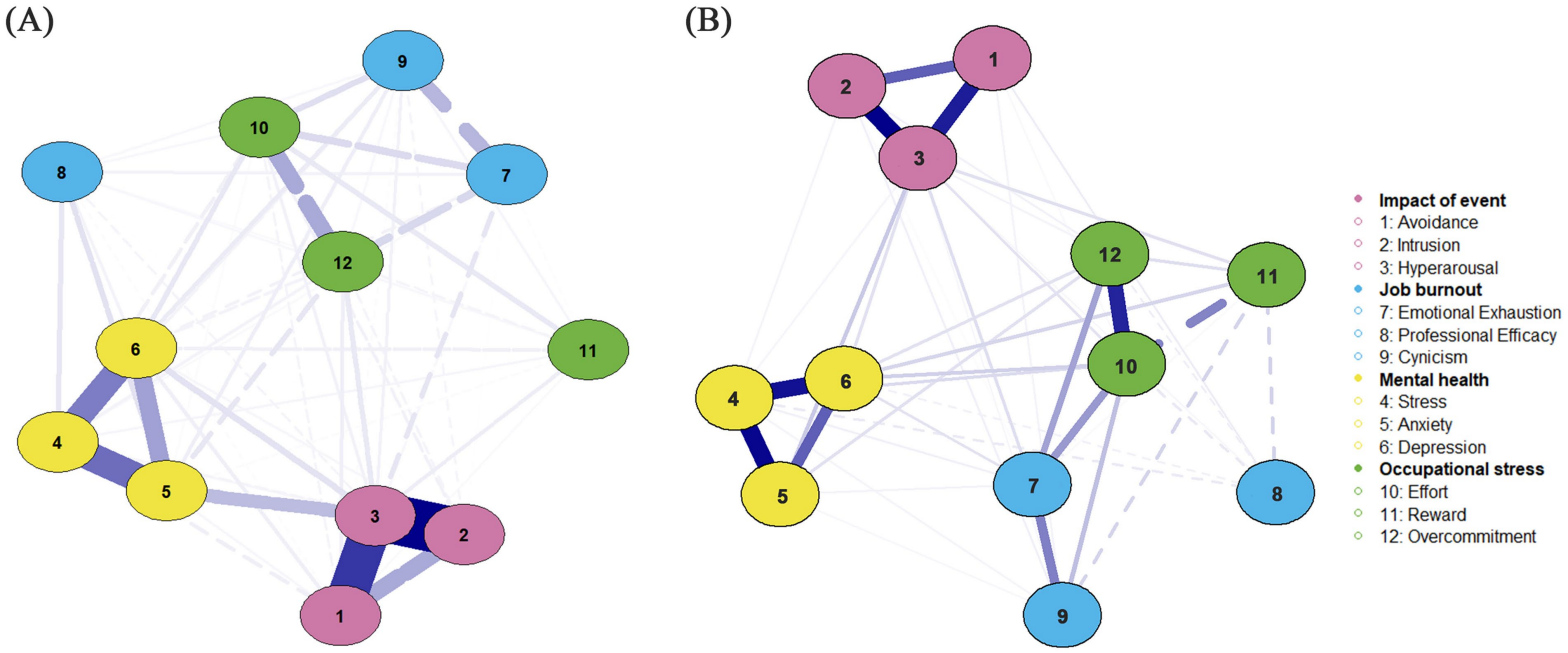
Network structure of PTSD symptoms, occupational stress, occupational burnout, and mental health among football referees. **(A)** Unadjusted network model. **(B)** Covariate-adjusted network model (controlled for age, sex, referee grade, working year, smoking, and drinking).

**Figure 2:**
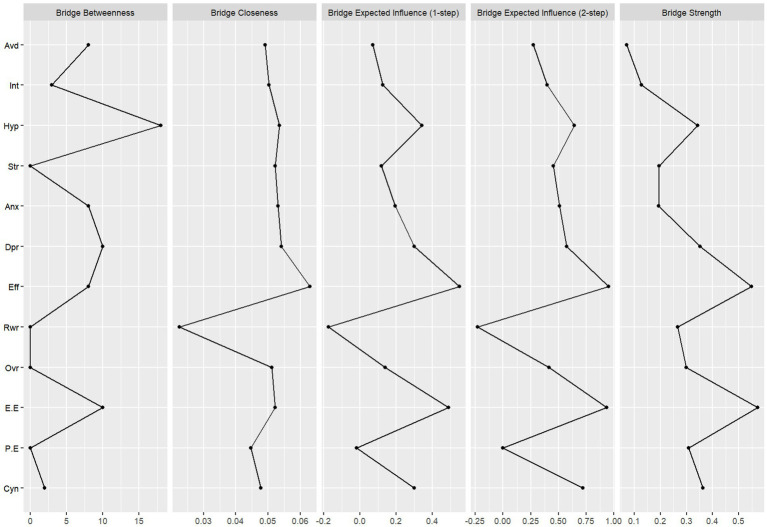
Bridge centrality indices for PTSD, occupational stress, burnout, and mental health dimensions. Avd, avoidance; Int, intrusion; Hyp, hyperarousal; Str, stress; Anx, anxiety; Dpr, depression; Eff, effort; Rwr, reward; Ovr, overcommitment; E. E, emotional exhaustion; P. E, professional efficacy; Cyn, cynicism.

**Figure 3:**
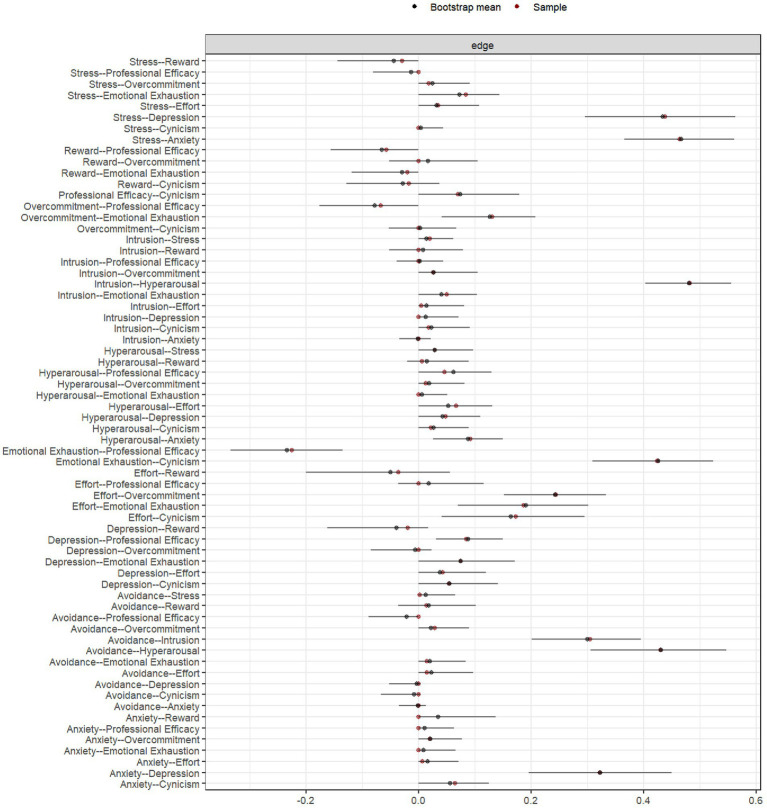
Bootstrap 95% confidence intervals for edge weights in the psychological symptom network.

Figure 1 presents the network model of event impact, occupational stress, occupational burnout, and mental health. The network consists of 12 nodes and 66 non-zero edges. Figure 1A shows the unadjusted model, while Figure 1B displays the covariate-adjusted model. Comparison of the two reveals that even after adjusting for covariates, Hyperarousal, Avoidance, and Intrusion remained significantly positively connected. Strong positive associations also persisted among effort, emotional exhaustion, and overcommitment. Several dimensions within occupational stress and occupational burnout maintained robust links with both PTSD and mental health variables, suggesting they may function as key bridge nodes. Centrality analysis revealed that hyperarousal had the highest expected influence (EI = 1.1913), indicating the strongest net influence on other variables in the network. This was followed by stress (0.8003), depression (0.7720), and anxiety (0.6020). For strength and betweenness metrics, hyperarousal and emotional exhaustion had the highest values, suggesting strong overall connectivity and frequent positioning along the shortest paths between other nodes, indicating potential mediating roles in the network. In terms of closeness centrality, emotional exhaustion (1.1839) and stress (0.6360) ranked highest, suggesting that these nodes maintain shorter average path lengths to other variables and may therefore facilitate more efficient transmission of psychological states. In contrast, nodes such as reward, professional efficacy, and overcommitment showed negative or low values across all four centrality indices, placing them at the periphery of the network—more likely to be consequence variables rather than influential ones (Table 2). Bridge centrality analysis (Figure 2) further identified effort, emotional exhaustion, and hyperarousal as nodes with consistently high values across multiple bridging metrics, indicating their potential role in connecting distinct symptom domains. Conversely, reward and professional efficacy scored low across most bridge indices, suggesting a limited role in cross-domain symptom propagation. Network stability analysis showed that the expected influence centrality index had a CS-coefficient of 0.75—exceeding the 0.50 threshold—indicating high interpretive reliability and test–retest consistency. As illustrated in Figure 3, the confidence intervals for most edge weights encompassed the original sample estimates, further supporting the robustness of the network estimation. Finally, network comparisons by gender revealed no statistically significant differences in network structure, edge weights, global strength, or node centrality between male and female referees (*p* > 0.05).

**Table 2:** Centrality indices of PTSD, occupational stress, occupational burnout, and mental health symptoms in the network model.

Variables	Betweenness	Closeness	Strength	Expected influence
Avoidance	−0.8764	−0.6933	−0.1587	0.2416
Intrusion	−0.8764	−0.5615	0.1617	0.4602
Hyperarousal	1.9282	−0.0974	1.2338	1.1913
Stress	−0.3506	0.6360	0.8520	0.8003
Anxiety	0.1753	0.4964	0.3697	0.6020
Depression	−0.1753	0.4443	0.7414	0.7720
Effort	0.5259	0.6353	−0.1423	0.0897
Reward	−0.8764	−2.6397	−2.1510	−1.9139
Overcommitment	−0.8764	−0.3517	−1.0130	−0.6399
Emotional exhaustion	1.5776	1.1839	1.1548	0.0461
Professional efficacy	0.7012	0.4585	−1.0026	−1.8898
Cynicism	−0.8764	0.4890	−0.0457	0.2403

### 3.3 Set-ESEM

The model fit indices for the set-ESEM indicated an acceptable model fit: RMSEA = 0.063 < 0.08, SRMR = 0.054 < 0.08, *p* < 0.001, with a 90% confidence interval [0.061, 0.065], which does not include zero. These results support the adequacy of the model fit. Factor loadings ≥ 0.30 were considered to have practical significance. As shown in the Geomin-rotated factor loading results for the four latent variables (Supplementary Table 2), all items from the PTSD and mental health scales showed significant positive loadings above 0.50, indicating good structural validity. The ERI scale included a small number of reverse-coded items (e.g., ERI10–ERI13, ERI20), which demonstrated negative but significant loadings, confirming satisfactory construct validity. For the MBI-GS, several items exhibited non-significant loadings, suggesting the need for further differentiation of its multidimensional structure in future modeling. Among the control variables, only referee grade showed a significant negative effect on the main latent constructs, while all other covariates had no significant influence. Overall, the four-factor structure demonstrated strong theoretical interpretability and structural distinctiveness, supporting the conceptualization of PTSD, psychological distress, occupational stress, and occupational burnout as four core and distinguishable dimensions for subsequent analysis. Factor correlation results revealed significant positive correlations between PTSD and both occupational stress (*r* = 0.473, *p* < 0.05) and mental health (*r* = 0.506, *p* < 0.05). Occupational stress was also positively associated with mental health (*r* = 0.504, *p* < 0.05). However, occupational burnout showed low and non-significant correlations with the other three constructs (|*r*| < 0.10, *p* ≥ 0.05), indicating limited statistical association.

### 3.4 SEM and mediation analysis

The results of the SEM demonstrated that PTSD significantly and positively predicted occupational stress (*B* = 0.003, *p* < 0.001), occupational burnout (*B* = 0.013, *p* < 0.001), and mental health (*B* = 0.528, *p* < 0.001). All path coefficients were statistically significant, thereby supporting Hypothesis 1. Both occupational stress and occupational burnout negatively predicted mental health (*B* = 39.826, *p* < 0.001; *B* = 8.443, *p* < 0.001). Table 3 presents the results of the mediation analysis examining the roles of occupational stress, occupational burnout, and their subdimensions in the relationship between PTSD and mental health. The findings indicate that occupational stress, occupational burnout, effort, overcommitment, emotional exhaustion, and cynicism each served as partial mediators between PTSD and mental health, with indirect effects accounting for 19.626, 24.744, 24.969, 10.058, 30.873, and 26.088% of the total effect, respectively. However, the mediating effects of reward and professional efficacy were not statistically significant. These results support Hypotheses 2, 2a, 2c, 3, 3a, and 3c, indicating that while occupational stress and occupational burnout function as mediators in the PTSD–mental health pathway, not all of their subdimensions necessarily contribute to this mechanism. Furthermore, chain mediation analyses were conducted for the significant mediating variables to explore their sequential indirect effects between PTSD and mental health. As shown in Table 4, the 95% confidence intervals for five mediation paths did not include zero, indicating that portions of Hypothesis 4 were also supported. In the foregoing analyses, set-ESEM reports zero-order latent correlations with cross-loadings permitted. Although occupational burnout shows low and non-significant associations with the other three constructs (|*r*| < 0.10, *p* ≥ 0.05), the mediation model estimates conditional (partial) relations after controlling for other constructs and measurement error. Consequently, even when the corresponding zero-order correlations are small or non-significant, the indirect effects may still be statistically significant.

**Table 3:** Results of mediation analysis.

Paths	Effect	Boot SE	BootLLCI	BootULCI	Proportion of effect	*p*
PTSD → Occupational stress → Mental health	0.156	0.033	0.046	0.174	19.626%	0.000
PTSD → Effort → Mental health	0.198	0.033	0.086	0.215	24.969%	0.000
PTSD → Reward → Mental health	0.003	0.011	−0.016	0.032	0%	0.798
PTSD → Overcommitment → Mental health	0.080	0.021	0.022	0.107	10.058%	0.000
PTSD → Occupational burnout → Mental health	0.196	0.027	0.095	0.203	24.744%	0.000
PTSD → Emotional exhaustion → Mental health	0.245	0.035	0.118	0.254	30.873%	0.000
PTSD → Professional efficacy → Mental health	0.002	0.004	−0.002	0.016	0%	0.657
PTSD → Cynicism → Mental health	0.207	0.032	0.099	0.226	26.088%	0.000

**Table 4:** Results of chain mediation analysis.

Paths	Effect	Boot SE	BootLLCI	BootULCI	*z*	*p*
PTSD → Occupational stress → Occupational burnout → Mental health	0.041	0.008	0.015	0.046	5.033	0.000
PTSD → Effort → Emotional exhaustion → Mental health	0.085	0.017	0.031	0.098	4.858	0.000
PTSD → Effort → Cynicism → Mental health	0.070	0.015	0.027	0.084	4.817	0.000
PTSD → Overcommitment → Emotional exhaustion → Mental health	0.051	0.011	0.019	0.063	4.559	0.000
PTSD → Overcommitment → Cynicism → Mental health	0.030	0.009	0.007	0.042	3.342	0.001

## 4 Discussion

To the best of our knowledge, this is the first study to integrate network analysis, set-ESEM, and mediation modeling to investigate the complex relationships and underlying mechanisms among post-pandemic PTSD, occupational stress, occupational burnout, and mental health in football referees. The results demonstrate that the network model comprising subdimensions of PTSD, occupational stress, occupational burnout, and mental health is highly reliable. Notably, subdimensions such as effort and emotional exhaustion exhibited strong bridging roles across symptom clusters. The set-ESEM findings revealed significant positive associations between PTSD, occupational stress, and mental health, whereas occupational burnout showed no significant correlations with the other three latent factors. However, mediation analyses were consistent with both occupational stress and occupational burnout operating as statistical indirect associations but also as sequential (chain) mediators in the relationship between PTSD and mental health. These findings suggest that occupational burnout, particularly through emotional exhaustion and functional decline, may function as a crucial bridge in the psychological stress pathway. Furthermore, we identified that effort, overcommitment, emotional exhaustion, and cynicism were statistically involved in both independent and chain indirect paths between PTSD and mental health, while reward and professional efficacy did not exhibit significant mediating effects. This study delineates patterns consistent with pathways by which PTSD may relate to mental health, offering both theoretical and practical implications for the prevention and intervention of PTSD and related psychological issues among football referees. The findings also provide empirical support for the development of targeted mental health strategies, contributing to the broader advancement of the football profession.

This study found a notably high prevalence of PTSD among football referees, with moderate-to-severe symptoms present in 26.2% of participants—consistent with findings from previous research. Earlier reviews have reported that approximately 18.75% of healthcare workers experienced PTSD during the early stages of the pandemic (31), with post-pandemic prevalence rates rising further—reaching 27% in women and 26% in men (32). The prevalence observed in this study aligns closely with these figures. Further analysis revealed that PTSD was significantly associated with poorer mental health, a finding that is consistent with extensive domestic and international literature. On one hand, persistent hyperarousal, recurrent intrusive memories, and avoidance behaviors resulting from trauma exposure deplete psychological resources and may be associated with higher anxiety, depression, and stress (33). On the other hand, the SARS-CoV-2 pandemic was associated with reduced physical activity and disrupted work routines for referees. Concurrently, heightened fear of infection, diminished family and social support, and increased financial pressure further compounded the risk of poor mental health outcomes (34). This study underscores that football referees, as a unique occupational group, were especially vulnerable to the pandemic’s disruption of professional routines, training schedules, and competitive stability—factors that contributed to trauma exposure and resource depletion. These conditions collectively may have heightened the susceptibility to PTSD and mental health problems within this population. Importantly, even after the pandemic has subsided, the lingering psychological consequences may be associated with reduced professional performance, health, and daily functioning. Therefore, this research highlights the urgent need to develop targeted intervention strategies aimed at enhancing psychological resilience and strengthening preparedness for future public health crises among football referees.

Network analysis revealed significant associations between subdimensions of occupational stress and key dimensions of both PTSD and mental health, particularly with respect to effort and overcommitment. Mediation analyses were additionally consistent with occupational stress—and specifically effort and overcommitment—participating in indirect associations between PTSD and mental health, in line with prior research. The SARS-CoV-2 pandemic was accompanied by an increased prevalence of PTSD among football referees. PTSD symptoms—such as heightened vigilance and avoidance behaviors—may impair referees’ ability to adapt to professional demands. Additionally, the prolonged suspension of sporting activities was associated with increased occupational stress during the initial return to work, requiring referees to exert considerable effort, which may be associated with higher anxiety and depressive symptoms (16). These findings align with stressor theory, which posits that psychological and physiological symptoms are likely to emerge when individuals perceive their external environment as highly stressful (15). Studies on athletes have similarly shown elevated anxiety levels during the early phases of competition following the pandemic (35). Therefore, this study highlights the importance of appropriately managing referees’ workloads and ensuring timely access to psychological and social support resources during the resumption of sports events.

Furthermore, this study found that PTSD was associated with poorer mental health indirectly via higher occupational burnout, particularly through the subdimensions of emotional exhaustion and cynicism—a finding consistent with previous research. A study on German emergency responders revealed that overcommitment and resource imbalance were associated with to emotional exhaustion, which was in turn associated with depressive symptoms, with emotional exhaustion playing a stronger mediating role than depersonalization (36). Similarly, during the pandemic, PTSD symptoms among healthcare workers were found to directly predict occupational burnout, which subsequently exacerbated psychological distress (37). These findings are in strong agreement with the conclusions of the present study. Accordingly, this research reinforces the theoretical basis for a PTSD–occupational burnout–mental health pathway, wherein emotional exhaustion and depersonalization serve as key mechanisms. PTSD is often accompanied by hyperarousal, avoidance, and intrusive symptoms, which are theorized to deplete emotional and cognitive resources. In the context of sustained resource loss, individuals may be more likely to experience emotional exhaustion. To prevent further depletion, they may adopt defensive coping strategies such as disengagement or cynicism—manifested through emotional detachment or reduced professional efficacy—to distance themselves from high-pressure environments (38). While these strategies may temporarily reduce perceived strain, they may be associated with lower individual’s capacity to cope with stress, thereby intensifying anxiety and depression and ultimately further deteriorating mental health. These findings are consistent with the core assumptions of the Job Demands–Resources model (19). Notably, the set-ESEM results in this study indicated that occupational burnout was not significantly associated with PTSD, occupational stress, or mental health at the latent structural level. However, significant effects emerged in both network analysis and mediation models. This discrepancy may reflect methodological differences between ESEM and regression-based approaches in handling measurement error, dimensional complexity, and structural assumptions (30). Whereas ESEM offers a structural perspective on the multidimensional nature of occupational burnout, the mediation model reveals the functional role of occupational burnout as a “bridge mechanism” within the psychological stress process.

This study further confirmed the sequential mediating role of occupational stress and occupational burnout in the relationship between PTSD and mental health. Specifically, the findings suggest that following exposure to traumatic events—such as the health threats and occupational disruptions caused by the SARS-CoV-2 pandemic—football referees tend to experience a marked increase in perceived occupational stress. This is particularly evident in the effort and overcommitment dimensions of the ERI model (39). PTSD symptoms may be associated with difficulties in work adaptation, heightening subjective workload and psychological effort (40). Compounding this stress, the temporary loss of job-related resources in the post-pandemic period—such as reduced income, limited physical training, and suspended competitions—exacerbates the strain. Occupational stress is consistently associated with occupational burnout. Prolonged exposure to high stress levels may deplete emotional regulation resources, and is associated with symptoms of emotional exhaustion and cynicism, thereby increasing the risk of occupational burnout (41). These findings are consistent with prior studies, which have also observed similar stress–burnout–psychological distress mechanisms among healthcare professionals during the pandemic (42). It is important to note that occupational burnout not only arises as a consequence of stress but may, in turn, undermine an individual’s coping capacity, thereby intensifying symptoms of anxiety, depression, and other mental health issues. This mechanism is especially pronounced in high-responsibility, high-intensity work environments such as football refereeing, where the demands of returning to work, readjusting skills, and meeting public expectations after the pandemic amplify the chain reaction. Therefore, intervention strategies should simultaneously target both occupational stress management and occupational burnout prevention. Reducing structural imbalances in work demands and enhancing individual coping resources at the source may effectively disrupt this sequential transmission pathway, thereby mitigating the mental health burden associated with PTSD.

## 5 Strengths and limitations

This study is the first to integrate network analysis, set-ESEM, SEM, and mediation analysis to systematically elucidate how PTSD influences the mental health of football referees through occupational stress and occupational burnout. It represents the first empirical investigation of these mechanisms within the context of Chinese football referees. By focusing on football referees—a highly specialized yet underrepresented occupational group—this research provides a theoretical foundation for psychological interventions in professional sports settings.

Nonetheless, several limitations should be acknowledged. First, the cross-sectional design limits the ability to draw causal inferences regarding the relationships among variables. The dynamic, time-dependent nature of PTSD, occupational stress, occupational burnout, and mental health could not be captured. Future longitudinal studies are needed to validate the directional pathways and mechanisms proposed in this research. Second, although the sample included referees from multiple provinces across China, all data were collected via self-report measures, which may introduce selection bias and recall bias. Future studies should incorporate objective indicators (e.g., fNIRS assessments) or third-party evaluations to further corroborate the proposed mechanisms. Finally, while this study controlled for several key demographic covariates (e.g., age, gender, referee certification level), it did not account for other potentially influential factors such as perceived social support, socioeconomic status, media scrutiny, split-second decision pressure, and public accountability. Future research should aim to include a broader set of covariates or examine the moderating roles of these variables in the psychological mechanisms of football referees to enhance the robustness and generalizability of the findings.

## 6 Conclusion

This study systematically examined the complex relationships and underlying mechanisms among PTSD, occupational stress, occupational burnout, and mental health in Chinese football referees following the SARS-CoV-2 pandemic. The findings demonstrate that both occupational stress and occupational burnout serve not only as independent mediators but also as sequential mediators in the pathway from PTSD to mental health outcomes. The subdimensions of effort, overcommitment, emotional exhaustion, and cynicism were identified as critical components within this mechanism. This research contributes to the theoretical understanding of how PTSD affects mental health in the context of public health crises, particularly within the understudied population of football referees. Moreover, it provides a robust empirical foundation for developing targeted psychological interventions and support policies within the field of professional sports officiating. Future research should pursue longitudinal designs to clarify causal pathways and explore the moderating roles of protective factors—such as social support and self-efficacy—in mitigating the psychological impact of traumatic exposure.

## Data Availability

The raw data supporting the conclusions of this article will be made available by the authors, without undue reservation.
