# Supplementary material for: The relationship between post-traumatic stress disorder, occupational stress, occupational burnout, and mental health in football referees: a national cross-sectional survey in China
Source: Front Public Health. 2025 Sep 8;13:1647115. doi: 10.3389/fpubh.2025.1647115 (PMC12450910; doi:10.3389/fpubh.2025.1647115)
Supplement: Supplementary file 1 [file Table_1.DOCX]

Supplementary Material

**Supplementary Table 1.** Results of correlation analysis among variables.

**Supplementary Table 2.** Geomin-rotated factor loadings of all items in the four-factor ESEM model (significant at the 5% level).

**Supplementary Table 1.** Results of correlation analysis among variables.

| **Variables** | **1** | **2** | **3** | **4** | **5** | **6** | **7** | **8** | **9** | **10** | **11** | **12** | **13** | **14** | **15** | **16** |
| --- | --- | --- | --- | --- | --- | --- | --- | --- | --- | --- | --- | --- | --- | --- | --- | --- |
| 1. PTSD | 1 |  |  |  |  |  |  |  |  |  |  |  |  |  |  |  |
| 2. Avoidance | 0.937** | 1 |  |  |  |  |  |  |  |  |  |  |  |  |  |  |
| 3. Intrusion | 0.936** | 0.787** | 1 |  |  |  |  |  |  |  |  |  |  |  |  |  |
| 4. Hyperarousal | 0.938** | 0.822** | 0.845** | 1 |  |  |  |  |  |  |  |  |  |  |  |  |
| 5. Occupational stress | 0.385** | 0.341** | 0.357** | 0.395** | 1 |  |  |  |  |  |  |  |  |  |  |  |
| 6. Effort | 0.470** | 0.417** | 0.436** | 0.477** | 0.848** | 1 |  |  |  |  |  |  |  |  |  |  |
| 7. Reward | -0.038 | -0.025 | -0.042 | -0.044 | -0.550** | -0.138* | 1 |  |  |  |  |  |  |  |  |  |
| 8. Overcommitment | 0.363** | 0.328** | 0.341** | 0.357** | 0.383** | 0.482** | -0.037 | 1 |  |  |  |  |  |  |  |  |
| 9. Occupational burnout | 0.432** | 0.352** | 0.415** | 0.464** | 0.418** | 0.483** | -0.176** | 0.263** | 1 |  |  |  |  |  |  |  |
| 10. Emotional exhaustion | 0.482** | 0.428** | 0.462** | 0.470** | 0.519** | 0.586** | -0.133* | 0.448** | 0.615** | 1 |  |  |  |  |  |  |
| 11. Professional efficacy | 0.038 | 0.002 | 0.036 | 0.084 | -0.014 | -0.019 | -0.085 | -0.125* | 0.606** | -0.195** | 1 |  |  |  |  |  |
| 12. Cynicism | 0.444** | 0.370** | 0.431** | 0.461** | 0.456** | 0.551** | -0.135* | 0.343** | 0.726** | 0.686** | 0.013 | 1 |  |  |  |  |
| 13. Mental health | 0.591** | 0.509** | 0.548** | 0.624** | 0.480** | 0.526** | -0.144** | 0.372** | 0.545** | 0.590** | 0.063 | 0.557** | 1 |  |  |  |
| 14. Stress | 0.571** | 0.499** | 0.530** | 0.593** | 0.459** | 0.506** | -0.160** | 0.369** | 0.495** | 0.574** | 0.023 | 0.520** | 0.963** | 1 |  |  |
| 15. Anxiety | 0.563** | 0.484** | 0.516** | 0.604** | 0.424** | 0.491** | -0.089 | 0.361** | 0.513** | 0.543** | 0.065 | 0.532** | 0.948** | 0.872** | 1 |  |
| 16. Depression | 0.553** | 0.469** | 0.519** | 0.588** | 0.489** | 0.504** | -0.158** | 0.330** | 0.552** | 0.567** | 0.099 | 0.541** | 0.947** | 0.867** | 0.843** | 1 |

**Supplementary Table 2.** Geomin-rotated factor loadings of all items in the four-factor ESEM model (significant at the 5% level).

| **Items** | **1** | **2** | **3** | **4** |
| --- | --- | --- | --- | --- |
| IES1 | 0.729* | 0.037 | 0.011 | -0.046 |
| IES2 | 0.540* | -0.096 | 0.056 | -0.013 |
| IES3 | 0.813* | -0.046 | -0.104 | 0.029 |
| IES4 | 0.802* | -0.100* | -0.005 | -0.005 |
| IES5 | 0.774* | 0.061 | 0.010 | -0.036 |
| IES6 | 0.381* | 0.108 | 0.146* | -0.130* |
| IES7 | 0.759* | -0.018 | -0.046 | -0.045 |
| IES8 | 0.684* | 0.007 | 0.011 | -0.052 |
| IES9 | 0.491* | 0.045 | 0.017 | -0.093 |
| IES10 | 0.577* | 0.127 | 0.003 | 0.006 |
| IES11 | 0.494* | -0.031 | 0.054 | -0.079 |
| IES12 | 0.545* | 0.077 | 0.027 | -0.073 |
| IES13 | 0.798* | -0.021 | -0.064 | -0.043 |
| IES14 | 0.716* | -0.027 | 0.060 | 0.072 |
| IES15 | 0.688* | 0.185* | 0.030 | 0.031 |
| IES16 | 0.647* | 0.104 | 0.065 | 0.119* |
| IES17 | 0.543* | 0.210* | 0.126 | -0.002 |
| IES18 | 0.776* | 0.061 | 0.011 | 0.068* |
| IES19 | 0.744* | 0.059 | 0.053 | 0.053 |
| IES20 | 0.769* | 0.107* | 0.026 | 0.059* |
| IES21 | 0.726* | 0.081 | -0.002 | 0.148* |
| IES22 | 0.585* | 0.073 | 0.019 | -0.118* |
| DASS1 | 0.271* | 0.508* | -0.047 | -0.039 |
| DASS2 | 0.202* | 0.656* | -0.038 | 0.077* |
| DASS3 | 0.053 | 0.712* | 0.041 | -0.110* |
| DASS4 | 0.000 | 0.839* | 0.017 | 0.005 |
| DASS5 | -0.027 | 0.852* | 0.039 | -0.068* |
| DASS6 | -0.028 | 0.705* | 0.051 | -0.144* |
| DASS7 | -0.003 | 0.760* | 0.041 | -0.041 |
| DASS8 | 0.107 | 0.551* | 0.105 | -0.051 |
| DASS9 | 0.066 | 0.639* | -0.042 | 0.013 |
| DASS10 | 0.119 | 0.653* | -0.098 | 0.043 |
| DASS11 | 0.039 | 0.678* | 0.046 | -0.104* |
| DASS12 | 0.106 | 0.733* | 0.001 | 0.047 |
| DASS13 | 0.028 | 0.740* | 0.017 | -0.014 |
| DASS14 | 0.142* | 0.704* | -0.015 | 0.035 |
| DASS15 | 0.139* | 0.581* | 0.101 | 0.018 |
| DASS16 | 0.086 | 0.635* | 0.063 | 0.005 |
| DASS17 | -0.034 | 0.796* | 0.066 | 0.033 |
| DASS18 | -0.033 | 0.778* | 0.078 | -0.036 |
| DASS19 | 0.051 | 0.708* | 0.057 | 0.042 |
| DASS20 | 0.102 | 0.684* | -0.059 | 0.045 |
| DASS21 | 0.045 | 0.671* | 0.001 | 0.068 |
| ERI1 | -0.016 | 0.035 | 0.667* | 0.073 |
| ERI2 | -0.021 | 0.129 | 0.575* | 0.037 |
| ERI3 | 0.039 | 0.071 | 0.593* | -0.068 |
| ERI4 | -0.067 | 0.127 | 0.625* | 0.027 |
| ERI5 | -0.008 | 0.068 | 0.659* | -0.044 |
| ERI6 | 0.145 | -0.081 | 0.630* | 0.112* |
| ERI7 | 0.157 | -0.165 | 0.488* | -0.034 |
| ERI8 | 0.120 | -0.087 | 0.459* | -0.020 |
| ERI9 | 0.164 | -0.173 | 0.533* | -0.020 |
| ERI10 | -0.199* | -0.074 | -0.407* | -0.068 |
| ERI11 | -0.042 | 0.005 | -0.501* | 0.008 |
| ERI12 | 0.027 | -0.059 | -0.620* | -0.050 |
| ERI13 | 0.067 | -0.048 | -0.635* | -0.100* |
| ERI14 | 0.153 | -0.158 | 0.531* | 0.026 |
| ERI15 | 0.011 | -0.007 | 0.530* | -0.007 |
| ERI16 | 0.131 | -0.164 | 0.466* | -0.019 |
| ERI17 | 0.182 | -0.185 | 0.480* | -0.034 |
| ERI18 | 0.022 | 0.035 | 0.603* | 0.004 |
| ERI19 | 0.016 | 0.019 | 0.412* | -0.079 |
| ERI20 | 0.068 | 0.183* | -0.359* | 0.101* |
| ERI21 | 0.002 | -0.047 | 0.523* | -0.069 |
| ERI22 | 0.027 | 0.045 | 0.306* | -0.185* |
| ERI23 | 0.127 | 0.096 | 0.293* | -0.154* |
| MBI1 | 0.034 | 0.309* | 0.329* | -0.152* |
| MBI2 | 0.127* | 0.278* | 0.375* | -0.139* |
| MBI3 | -0.035 | 0.254 | 0.449* | 0.040 |
| MBI4 | 0.051 | -0.051 | 0.103 | -0.621* |
| MBI5 | -0.139 | 0.342* | 0.468* | -0.036 |
| MBI6 | 0.092 | 0.387* | 0.395* | 0.021 |
| MBI7 | 0.017 | 0.283* | 0.447* | -0.023 |
| MBI8 | -0.045 | 0.277* | 0.481* | 0.004 |
| MBI9 | -0.003 | -0.012 | 0.041 | 0.740* |
| MBI10 | 0.056* | -0.037 | -0.030 | 0.894* |
| MBI11 | 0.050* | -0.021 | -0.018 | 0.954* |
| MBI12 | 0.007 | -0.063* | 0.014 | 0.873* |
| MBI13 | 0.022 | 0.009 | -0.003 | 0.915* |
| MBI14 | 0.010 | 0.017 | 0.081* | 0.916* |
| MBI15 | -0.033 | 0.012 | 0.026 | 0.937* |
| MBI16 | -0.035 | 0.201* | -0.007 | 0.779* |
| MBI17 | 0.030 | 0.204* | 0.298* | 0.014 |
| MBI18 | -0.103 | 0.232* | 0.420* | -0.023 |
| MBI19 | 0.064 | 0.134 | 0.432* | 0.090* |
| MBI20 | 0.133* | 0.110 | 0.429* | 0.196* |
| MBI21 | -0.008 | 0.279* | 0.480* | 0.087* |
| MBI22 | -0.016 | 0.189 | 0.433* | -0.150* |
| Sex | -0.108 | 0.208* | -0.086 | 0.015 |
| Age | 0.091 | -0.167 | 0.013 | -0.127* |
| Referee grade | -0.180* | 0.149 | -0.175 | 0.100 |
| Working year | 0.148 | -0.165 | 0.129 | -0.102 |
| Smoking | 0.026 | -0.117 | 0.126 | -0.003 |
| Drinking | 0.023 | -0.167* | 0.158* | -0.021 |
